# Thrombolysis vs Anticoagulation: Unveiling the Trade-Offs in Massive Pulmonary Embolism

**DOI:** 10.7759/cureus.52675

**Published:** 2024-01-21

**Authors:** Tamam Mohamad, Eyas Kanaan, Ikponmwosa J Ogieuhi, Anitte Shah Mannaparambil, Rubela Ray, Laith Wail Majed Al-Nazer, Hajra Munir Ahmed, Muzaffer Hussain, Narendar Kumar, Komal Kumari, Muhammad Nadeem, Sanvi Kumari, Giustino Varrassi

**Affiliations:** 1 Cardiovascular Medicine, Wayne State University, Detroit, USA; 2 Internal Medicine, Corewell Health, Grand Rapids, USA; 3 Physiology, University of Benin, Benin City, NGA; 4 General Medicine, Siberian State Medical University, Tomsk, RUS; 5 Public Health, University of Glasgow, Glasgow, GBR; 6 Internal Medicine, Bankura Sammilani Medical College and Hospital, Bankura, IND; 7 Medicine, University of Jordan, Amman, JOR; 8 Medicine, Al Zafer Hospital, Jeddah, SAU; 9 Medicine, Karachi Medical and Dental College, Karachi, PAK; 10 Internal Medicine, Burjeel Hospital, Abu Dhabi, ARE; 11 Medicine, NMC Royal Family Medical Centre, Abu Dhabi, ARE; 12 Medicine, Khyber Teaching Hospital, Peshawar, PAK; 13 Internal Medicine, Jinnah Sindh Medical University, Karachi, PAK; 14 Pain Medicine, Paolo Procacci Foundation, Rome, ITA

**Keywords:** venous thromboembolism, hemodynamic stability, massive pulmonary embolism, anticoagulation, thrombolysis, pulmonary embolism

## Abstract

Massive pulmonary embolism (MPE) is a severe form of venous thromboembolism (VTE) wherein enormous blood clots block the pulmonary arteries, resulting in substantial illness and death. Even with the progress made in diagnostic methods and treatments, the most effective approach for managing MPE is still a topic of considerable discussion. This study examines the delicate equilibrium between thrombolysis and anticoagulation in managing the problematic clinical situation posed by MPE, elucidating the compromises linked to each strategy. The genesis of MPE lies in the pathophysiology of VTE, when blood clots that originate from deep veins in the lower legs or pelvis move to the pulmonary vasculature, leading to an abrupt blockage. This obstruction leads to a series of hemodynamic alterations, such as elevated pulmonary vascular resistance, strain on the right ventricle, and compromised cardiac output, finally resulting in cardiovascular collapse. The seriousness of MPE is commonly categorized according to hemodynamic stability, with significant cases presenting immediate risks to patient survival. Traditionally, heparin has been the primary approach to managing MPE to prevent the spread of blood clots and their movement to other parts of the body. Nevertheless, there have been ongoing discussions regarding the effectiveness of thrombolysis, which entails the immediate delivery of fibrinolytic drugs to remove the blood clot. The use of thrombolysis in managing MPE is being reconsidered because of concerns over bleeding complications and long-term results despite its capacity to resolve the blocking clot quickly. This review rigorously analyzes the current body of evidence, exploring the intricacies of thrombolysis and anticoagulation in MPE. The focus is on evaluating the risk-benefit balance of each treatment option, considering aspects such as the patient's other medical conditions, hemodynamic stability, and potential long-term consequences. This review aims to clarify the complexities of the thrombolysis versus anticoagulation dilemma. It seeks to provide clinicians, researchers, and policymakers with a thorough understanding of the trade-offs in managing MPE. The goal is to facilitate informed decision-making and enhance patient outcomes.

## Introduction and background

Massive pulmonary embolism (MPE) is a severe form of venous thromboembolism (VTE) wherein huge blood clots suddenly block the pulmonary arteries, posing a life-threatening situation. This vascular event causes significant changes in blood flow, requiring immediate and effective medical treatments to reduce the risk of illness and death. This review aims to provide insight into the continuing controversy surrounding two key therapeutic options for addressing MPE: thrombolysis and anticoagulation. Choosing between one technique and another has significant repercussions, leading to extensive consequences for patient outcomes and healthcare practices [1]. To fully understand the importance of MPE, placing it within the wider scope of VTE is crucial. VTE refers to the occurrence of deep vein thrombosis (DVT) and pulmonary embolism (PE), which are both caused by the development of blood clots within the veins. DVT commonly starts in the deep veins of the lower limbs or pelvis. If not treated, these blood clots can break loose and travel to the lungs, causing PE [2]. MPE, the main focus of this review, refers to a specific type of PE where large blood clots block the pulmonary arteries. This commonly results in acute strain on the right ventricle of the heart and subsequent compromise of the body's blood circulation. The pathophysiological process that triggers MPE is complex and involves multiple factors. The combination of venous stasis, hypercoagulability, and vascular endothelial damage leads to blood clots in the deep veins. When these blood clots travel to the blood vessels in the lungs, they block the flow of blood, which hampers the exchange of gases and leads to a dramatic rise in resistance in the pulmonary blood vessels [2]. As a result, the right ventricle experiences greater resistance to blood flow, causing immediate stress on the right ventricle and perhaps leading to a sudden cardiovascular system failure.

The classification of MPE is frequently determined according to hemodynamic stability, with major cases presenting an imminent risk to the patient's survival. Historically, the primary focus of managing MPE has been the timely introduction of anticoagulation using unfractionated heparin (UFH) or low-molecular-weight heparin (LMWH). The goal is to prevent the further spread of blood clots and their movement to other parts of the body [3]. Although this method is successful in some instances, it has motivated clinicians to investigate alternate strategies that tackle the clot burden more intensely. Thrombolysis is a strategy that involves using fibrinolytic drugs to quickly break a blocking blood clot and restore blood flow in the lungs. Thrombolysis in MPE is a topic of dispute. Although the possibility of quickly resolving the clot that is causing the obstruction is clear, there are concerns about bleeding problems, particularly in patients who are unstable regarding their blood circulation. These concerns have sparked ongoing discussions among medical professionals [3]. To determine if thrombolytic therapy is appropriate, it is important to carefully consider the advantages of quickly dissolving blood clots compared to the potential dangers of bleeding issues. This evaluation should consider individual patient characteristics, existing health conditions, and the overall balance between the benefits and risks of this treatment. On the other hand, depending exclusively on anticoagulation has its restrictions. Anticoagulants hinder the continued growth of blood clots but may not adequately reduce the size of existing clots, which could result in ongoing problems with blood flow and negative effects [4]. The importance of evaluating treatment options beyond routine anticoagulation is highlighted by long-term concerns such as the risk of chronic thromboembolic pulmonary hypertension (CTEPH) and the potential impact on quality of life.

This review explores the complex field of MPE management by carefully analyzing the compromises linked to thrombolysis and anticoagulation. The objective is to analyze the available literature methodically, combine important discoveries, and present a thorough examination. This will enable clinicians, researchers, and policymakers to understand the complex therapeutic challenges associated with MPE cases. The main goal is to support well-informed decision-making, ensuring that the selected therapeutic method matches the specific needs of patients and maximizes long-term results. In the following sections, we will examine the underlying causes of MPE, closely examine the current methods used to manage MPE, investigate the mechanisms and effectiveness of thrombolysis, evaluate the conventional approach of anticoagulation, perform a comparative analysis, and uncover the advantages and disadvantages associated with each strategy. By conducting this investigation, we aim to provide significant knowledge that can influence future research efforts and therapeutic recommendations and eventually improve the treatment and results for those struggling with the intricacies of MPE.

## Review

Methodology

This section explains the detailed and systematic approach to conducting the comprehensive review titled "Thrombolysis vs Anticoagulation: Revealing the Trade-Offs in Massive Pulmonary Embolism." The process followed stringent academic criteria, guaranteeing the strength and reliability of the review. An extensive search of electronic databases, such as PubMed, MEDLINE, Embase, and Cochrane Library, was performed. The search included pertinent peer-reviewed journals, conference proceedings, and grey literature, covering the entire publishing period from the beginning till now. We employed a combination of MeSH terms and free-text keywords to encompass a wide range of literature on MPE, thrombolysis, and anticoagulation - a narrative synthesis methodology - and integrated information from various investigations. Thematic analysis was employed to discover prevalent patterns, differences, and points of agreement. Quantitative data were combined when appropriate, and subgroup analyses were conducted to investigate differences. Although the technique was rigorous, it is essential to acknowledge numerous limitations. Firstly, there is a possibility of publication bias in this review due to its heavy reliance on published material. Furthermore, the ever-changing nature of medical research may lead to the omission of new studies due to the limited search duration. Moreover, the intrinsic diversity in the designs of studies and the characteristics of patients may lead to variations in the synthesis of results. Since this review solely focuses on analyzing existing literature and does not involve any direct participation of human subjects, there was no need to seek ethics committee approval. The research complies with ethical standards prescribed by the Declaration of Helsinki and pertinent institutional rules. The rigorous methodology utilized in this evaluation guarantees a thorough and impartial synthesis of the current evidence regarding the contentious issue of thrombolysis versus anticoagulation in the management of MPE. Recognizing the limits showcases openness and prepares for a detailed analysis of the review's conclusions.

Pathophysiology of MPE

Comprehending the biology of MPE is crucial for physicians and researchers who are dealing with the intricacies of this potentially fatal illness. This section examines the complex mechanisms involved in MPE, including VTE, the movement of blood clots to the pulmonary blood vessels, and the effects on blood flow.

Overview of VTE

VTE is a term that includes both DVT and PE. It occurs due to the interaction of three factors known as Virchow's triad: venous stasis, hypercoagulability, and vascular endothelial damage. Venous stasis is when blood flow slows down, leading to the buildup of substances that cause blood clotting. Hypercoagulability refers to an abnormality in the blood clotting process, where an uneven sequence of events leads to the development of blood clots, with a tendency towards increased clot formation [5]. Vascular endothelial injury from trauma or inflammation further leads to the onset of thrombogenesis. DVT frequently acts as the precursor to PE, with blood clots primarily developing in the deep veins of the lower extremities or pelvis. These blood clots, composed of fibrin, platelets, and red blood cells, can become emboli. The prothrombotic milieu within the deep veins lays the setting for the migration of thrombi, creating a risk for pulmonary embolization [5]. The movement of blood clots to the blood vessels in the lungs indicates the shift from a confined DVT to a severe state of PE. Thrombi, which are blood clots, move from the deep veins, travel through the venous system, and reach the right side of the heart. The right atrium propels the emboli into the pulmonary arteries, where they become lodged in the lesser branches of the pulmonary vascular tree [6]. Thrombi can be found in many parts of the pulmonary arteries, such as the central pulmonary arteries, lobar arteries, and segmental branches. The degree of blockage in the pulmonary blood vessels depends on blood clots' size, quantity, and distribution [6]. Within the framework of MPE, these blood clots are considerable in size, frequently resulting in severe blockage and hindering blood circulation to significant areas of the pulmonary blood vessels.

Hemodynamic Consequences of MPE 

Substantial blood clots in the pulmonary arteries trigger a series of hemodynamic repercussions. Chief among these is a considerable increase in pulmonary vascular resistance (PVR). Thrombi block blood flow, causing increased resistance in the pulmonary circulation. This hinders the right ventricle's capacity to pump blood into the pulmonary arteries. The enhanced PVR places a sudden burden on the right ventricle (RV), which requires more effort to sustain pulmonary blood flow despite the increased resistance [7]. This tension can result in right ventricular impairment, which hinders the RV's capacity to circulate blood into the pulmonary system effectively. In severe instances, this can advance to right ventricular failure, worsening the hemodynamic instability linked to MPE. Due to the higher workload and reduced contractility, the right ventricular experiences a decrease in cardiac output [7]. The reduction in the amount of blood pumped by the heart reduces the ability of the body to receive adequate blood flow, resulting in reduced blood supply to the organs and possibly causing multiple organ failure. The systemic ramifications of MPE go beyond the circulation in the lungs, emphasizing the broader consequences of a localized blood clotting event. The physiological effects of MPE are evident in patients as symptoms such as difficulty breathing, chest discomfort, rapid heart rate, and low blood pressure. In severe instances, there may be a sudden cardiovascular system failure, requiring immediate action to restore blood flow to the lungs and reduce the burden on the right side of the heart [8]. To summarize, the pathophysiology of MPE involves the formation of blood clots in the deep veins, their movement to the blood vessels in the lungs, and the effects on blood flow. This comprehensive understanding is crucial for doctors navigating the diagnostic and therapeutic landscape of MPE, providing a platform for tailored therapies to minimize the morbidity and mortality associated with this severe condition.

Current landscape of MPE management

Effectively handling MPE requires a sophisticated and adaptable approach, prioritizing prompt intervention and tactics grounded in scientific data. This section thoroughly examines the present state of MPE care, highlighting the significance of immediate intervention, the implementation of established anticoagulation regimens, and the formation of trends in thrombolysis application.

Significance of Prompt Intervention

Swift and precise diagnosis is crucial for timely intervention in MPE. Nevertheless, the diagnosis might be difficult because of the varied and vague clinical symptoms, including difficulty breathing, chest discomfort, and unstable blood flow. Achieving a conclusive diagnosis necessitates using clinical assessment, imaging techniques, and biomarkers, emphasizing the need for a comprehensive and timely diagnostic strategy [8]. Efficient management requires a systematic approach that considers the patient's hemodynamic stability. The high-risk aspect of MPE, especially when there is hemodynamic compromise, requires prompt management to reduce the danger of cardiovascular collapse. Using risk stratification techniques, such as the Pulmonary Embolism Severity Index (PESI) and the European Society of Cardiology (ESC) risk assessment model, helps determine the urgency of therapeutic actions [9]. Advanced imaging techniques, such as computed tomography pulmonary angiography (CTPA) and ventilation-perfusion (V/Q) scans, are crucial for verifying the diagnosis and evaluating the degree of pulmonary vascular involvement. Combining modern imaging techniques and clinical factors enables prompt risk evaluation, providing a basis for selecting the most suitable therapeutic measures [9].

*Standard Anticoagulation Strategies* 

In the past, the primary approach to managing MPE has been using UFH or LMWH as the conventional anticoagulant treatment. These medicines obstruct the activity of thrombin and factor Xa, impeding the progression of blood clot formation and the release of emboli. The injection of UFH or LMWH has a double objective: to stop the advancement of existing blood clots and prevent the creation of new blood clots, thereby maintaining the patient's hemodynamic condition. Introducing oral anticoagulants and direct oral anticoagulants (DOACs) has significantly changed the approach to managing MPE [10]. Warfarin, rivaroxaban, apixaban, and dabigatran can be used instead of parenteral anticoagulants. These agents have the advantage of being administered orally, which is more convenient. Multiple studies have investigated the effectiveness and safety of DOACs, revealing that they produce similar or better results than conventional anticoagulation methods. Notwithstanding the prevalent utilization of anticoagulation, obstacles endure. Patients experiencing instability in their circulatory system may not experience immediate advantages from using anticoagulation alone, which leads to the investigation of more assertive treatment measures. Furthermore, the need for a customized and individualized strategy for anticoagulation is emphasized by the apprehensions related to bleeding problems, drug interactions, and the necessity for careful monitoring [10].

Emerging Trends in Thrombolysis Use

The current developments in MPE management involve reassessing the significance of thrombolysis, particularly in instances of high-risk MPE. Thrombolysis, which uses fibrinolytic drugs, aims to dissolve the blocking blood clots and restore lung blood flow. This technique seeks to overcome the constraints of using only anticoagulation, offering a more comprehensive therapeutic strategy for patients who are at a significant risk of experiencing cardiovascular collapse [11]. The increasing evidence supports thrombolysis as an effective treatment option in MPE. The Pulmonary Embolism Thrombolysis (PEITHO) study and other clinical trials have investigated the effectiveness and safety of thrombolysis in MPE. These trials have shown improvements in hemodynamic parameters and decreased the risk of hemodynamic decompensation [11]. The evidence is constantly developing as ongoing trials and observational studies improve guidelines and recommendations. Thrombolysis in MPE is dependent on meticulous patient selection. The decision-making process is guided by factors such as the severity of hemodynamic instability, the size and location of blood clots, and contraindications to fibrinolytic therapy. The growing inclination towards a personalized approach emphasizes the significance of carefully considering the advantages and disadvantages within the framework of the distinct clinical characteristics of every patient [12]. Thrombolysis has the potential to dissolve blood clots quickly, but it also carries the inherent danger of causing bleeding problems. Vigilant monitoring and careful analysis of the overall risk-benefit profile are necessary due to cerebral and systemic bleeding concerns. Ongoing research is focused on developing strategies to reduce bleeding risks, including dose optimization and implementing additional medicines [12]. Overall, the therapy of MPE is marked by a dynamic interaction between the significance of prompt intervention, established anticoagulation methods, and evolving patterns in thrombolysis utilization. Quick diagnosis and risk assessment are crucial in choosing the most appropriate treatment approach. Anticoagulation is a critical element of this individualized therapeutic approach, whereas thrombolysis is an increasingly complex and changing strategy used for high-risk patients of MPE. Clinicians are faced with the challenge of navigating a rugged terrain to optimize outcomes for persons dealing with the severe consequences of MPE as more research is gathered and guidelines are adjusted.

Thrombolysis: Mechanisms and efficacy

Thrombolysis, which involves giving fibrinolytic drugs, is a crucial treatment for MPE. This section thoroughly studies the mechanisms for fibrinolytic medicines, provides evidence for thrombolysis in MPE, and comprehensively analyzes this treatment's hemodynamic advantages and related dangers. An overview of commonly used thrombolytic agents is given in Table 1.

**Table 1:** Overview of thrombolysis agents

Fibrinolytic Agent	Mechanism of Action	Indications	Adverse Effects
Alteplase	Tissue plasminogen activator converting plasminogen to plasmin, which dissolves fibrin clots	Massive or submassive pulmonary embolism, acute ischemic stroke, myocardial infarction	Risk of bleeding, including intracranial hemorrhage, allergic reactions, hypotension, arrhythmias, fever, and nausea [9]
Tenecteplase	Modified form of alteplase with prolonged half-life and higher fibrin specificity	Massive or submassive pulmonary embolism, acute myocardial infarction	Similar to alteplase, with potential for reduced risk of bleeding complications [10]
Streptokinase	Forms a complex with plasminogen, converting it to plasmin and dissolving fibrin	Limited use due to increased risk of allergic reactions	Risk of bleeding, allergic reactions, hypotension, fever, nausea, and potential for neutralizing antibodies leading to reduced efficacy [11]

Fibrinolytic Agents and Their Actions

The fibrinolytic drugs used for MPE primarily fall into the category of plasminogen activators. Alteplase, streptokinase, and tenecteplase are drugs that facilitate the conversion of plasminogen to plasmin, which is the primary enzyme involved in fibrinolysis. Plasmin breaks down fibrin clots into smaller pieces and helps dissolve blood clots [13]. Alteplase, a commonly employed fibrinolytic drug, selectively attaches to fibrin present in the blood clot. Upon being bound, it triggers the activation of plasminogen, resulting in the production of plasmin. Plasmin enzymatically cleaves fibrin, forming fibrin degradation products, and aids in the disintegration of the blood clot. The fibrin specificity of alteplase improves its effectiveness in dissolving blood clots while reducing plasminogen activation throughout the body [14]. Tenecteplase, a derivative of alteplase, exhibits an improved affinity for fibrin and a prolonged duration of action. This alteration enhances the pharmacokinetics, enabling the delivery of a concentrated dose and decreasing the requirement for ongoing infusions. Tenecteplase's enhanced fibrin specificity increases its effectiveness in dissolving blood clots while potentially reducing the likelihood of bleeding throughout the body [14].

Supporting Evidence for Thrombolysis in MPE

The PEITHO experiment is a significant study that examines the use of thrombolysis in treating high-risk PE. This study conducted a randomized controlled trial to assess the effectiveness and safety of tenecteplase in combination with heparin, compared to a placebo in combination with heparin, in patients with normal blood pressure and exhibiting high-risk characteristics. The trial showed a notable decrease in the combined occurrence of death from any cause and hemodynamic decompensation with the administration of tenecteplase. However, this benefit came with the drawback of an elevated likelihood of experiencing severe bleeding [14]. Thrombolysis in MPE significantly improves the circulatory system, especially in individuals showing indicators of circulatory instability. Swift thrombus breakdown results in the reestablishment of blood flow in the lungs, relieving the elevated pulmonary vascular resistance typical of MPE. The resulting enhancement in the function of the right ventricle leads to an improved blood flow from the heart, reducing the likelihood of a sudden failure of the cardiovascular system and the failure of multiple organs [15]. Thrombolysis in MPE has been found to provide a significant advantage in quickly resolving right ventricular strain. Thrombolysis speeds up blood clot breakdown, which helps decrease the resistance on the right ventricle. This allows for a more effective pumping of blood into the lungs. Determining right ventricular strain is crucial in stabilizing hemodynamics and enhancing cardiovascular function. Thrombolysis can provide further long-term advantages by decreasing the likelihood of developing CTEPH [15]. Thrombolysis reduces the risk of remaining blood clots turning into organized fibrotic lesions, which can lead to CTEPH. The possible preventive effect of thrombolysis extends beyond the acute phase, highlighting its comprehensive influence [15].

Hemodynamic Risks and Considerations

Thrombolysis significantly improves blood flow, but it also has potential dangers. One of the main hazards is the possibility of experiencing bleeding issues. The systemic stimulation of plasminogen can cause fibrin degradation outside the blood clot, resulting in bleeding at different locations. Intracranial bleeding is a severe consequence that requires a cautious selection of patients and diligent monitoring [16]. Delivering thrombolysis in MPE requires a thorough evaluation of the risk-benefit profile. One must consider the risk of severe bleeding consequences in comparison to the need to address acute hemodynamic impairment promptly. Thrombolysis can be highly beneficial for patients who have high-risk factors like hypotension or shock. However, the risk-benefit ratio may not be favorable for patients with a reduced risk of decompensation [16]. Thrombolysis should be avoided in cases with contraindications, such as recent surgery, active bleeding, and specific comorbidities. Therefore, a customized and personalized strategy is required. The decision-making process is guided by meticulously evaluating patient-specific characteristics, including age, comorbidities, and bleeding risk. Close collaboration among cardiologists, pulmonologists, and hematologists is crucial to guarantee a thorough evaluation and well-informed judgment on thrombolytic therapy [17]. To reduce the likelihood of bleeding problems, other additional treatments might be used simultaneously, such as thrombolysis. Administering antifibrinolytic drugs, like tranexamic acid, can reduce systemic bleeding by preventing the breakdown of fibrin. It is crucial to closely monitor coagulation markers, hemoglobin levels, and vital signs to recognize and manage bleeding issues rapidly.

Prospects for Advancements in Thrombolysis Research

Research is now being conducted to improve the effectiveness of thrombolytic medicines in treating MPE, leading to changes in the landscape of thrombolysis. Investigations are being undertaken on novel drugs that have enhanced fibrin specificity, longer half-lives, and decreased bleeding hazards. These improvements aim to improve the effectiveness of thrombolysis treatment while reducing the related problems. To acknowledge the diversity within the MPE community, future research efforts are expected to investigate customized strategies for distinct subgroups [17]. Patient characteristics, including age, comorbidities, and the size and location of thrombi, may guide the selection of thrombolytic therapy or alternative interventions. The objective of personalized medicine techniques is to enhance results by tailoring therapy strategies to match the specific profiles of individual patients. Investigating combination therapies, which involve the integration of thrombolysis with various therapeutic techniques, is a promising area for future research [18]. Research studies are being conducted to explore the combined benefits of thrombolysis with catheter-directed therapies, surgical embolectomy, or innovative anticoagulant methods. The goal is to find comprehensive and multimodal approaches to managing MPE. Fibrinolytic drugs selectively target the blood clot, significantly improving the circulatory system of patients with high-risk PE. The existing body of evidence, as demonstrated by studies such as the PEITHO trial, emphasizes the potential benefits of thrombolysis while acknowledging the inherent hazards, particularly bleeding problems. Advancements in research are leading to the improvement of thrombolytic medicines, a more individualized approach to selecting patients, and the investigation of combination therapy. These developments can significantly impact the future of thrombolysis in the complex and ever-changing field of MPE management.

Anticoagulation: Traditional approach

Anticoagulation is widely considered the fundamental treatment for managing MPE. This section thoroughly examines the conventional method of anticoagulation, explaining the function of anticoagulants in MPE, investigating the drawbacks of relying solely on anticoagulation, and analyzing the consequences of unsatisfactory results and long-term factors.

Role of Anticoagulants in MPE

Anticoagulants are crucial in managing MPE by stopping the spread of blood clots in the lungs' blood vessels. Heparins, including unfractionated and low-molecular-weight forms, function as indirect anticoagulants by augmenting the efficacy of antithrombin, a naturally occurring inhibitor of clotting factors. This inhibition hinders the blood clotting process, limiting the production of more blood clots and lowering the chances of a blood clot traveling to another part of the body. According to medical guidelines, anticoagulation is the recommended treatment for hemodynamically stable MPE cases [18]. Hemodynamically stable patients usually have milder symptoms, which allows for a less intense treatment approach. Anticoagulation is used to treat the immediate blood clotting event while reducing the chances of bleeding complications linked to more intrusive treatments. Introducing oral anticoagulants and DOACs has broadened the range of available anticoagulation choices. Warfarin, a substance that inhibits the action of vitamin K, has been used for a long time as an oral anticoagulant. However, newer drugs known as DOACs, including rivaroxaban, apixaban, and dabigatran, provide alternative options with more predictable effects on the body's absorption, distribution, metabolism, and excretion, as well as more straightforward instructions for dosage [18]. These medications offer convenient and ambulatory treatment options for qualified patients.

Limitations of Anticoagulation Alone

Although anticoagulation successfully prevents the spread of blood clots, it is inefficient in reducing the amount of existing blood clots in the pulmonary blood vessels. When dealing with MPE, especially when blood clots are large and causing problems with blood flow, relying solely on anticoagulation may lead to less-than-ideal results. The prolonged presence of blocking blood clots can result in ongoing instability of blood flow and hinder the overall effectiveness of treatment [19]. Solely administering anticoagulation may not completely eradicate the possibility of developing CTEPH, a lasting outcome of unresolved blood clots in the lungs. While anticoagulation can stop the further spread of blood clots, it may not prevent the transformation of remaining clots into fibrotic lesions, leading to CTEPH [19]. This emphasizes the significance of contemplating alternate or supplementary therapy approaches. For MPE instances with a high risk, where patients show evidence of hemodynamic compromise or are at immediate risk of circulatory collapse, using only anticoagulation may result in worse-than-ideal outcomes. The prolongation in resolving the accumulation of blood clots and restoring blood flow in the lungs increases the likelihood of adverse outcomes, underscoring the necessity for more assertive treatments, such as thrombolysis or surgical embolectomy, in specific high-risk situations [19].

Suboptimal Outcomes and Long-Term Considerations

Anticoagulation as the primary treatment method in MPE may result in extended hospital stays, mainly when the patient's hemodynamic stability takes a long time to improve. Extended periods of hospitalization lead to higher usage of healthcare resources, requiring continuous monitoring, imaging examinations, and supportive care interventions. Optimizing treatment options is emphasized by the effect on healthcare systems and patient quality of life [20]. The difficulties in attaining therapeutic anticoagulation may hinder reaching optimal outcomes. Warfarin, a frequently prescribed oral anticoagulant, necessitates meticulous monitoring of international normalized ratio (INR) values to sustain therapeutic anticoagulation. Monitoring can be arduous, and striking the right balance between limiting the formation of blood clots and avoiding bleeding problems is a clinical difficulty [20]. The need for prolonged anticoagulation in MPE gives rise to worries over the accompanying dangers of bleeding. The risk of bleeding problems is increased when anticoagulants are used for an extended period, particularly in cases of chronic comorbidities or recurrent embolic episodes. Deliberating the advantages of ongoing anticoagulation in preventing recurring thromboembolism compared to the potential for bleeding problems necessitates meticulous evaluation and continuous risk assessment [20]. The consequences of prolonged anticoagulation go beyond medical factors, affecting the overall well-being of patients with MPE. Chronic therapy requires strict adherence to prescribed pharmaceutical regimens, regular monitoring, and making necessary lifestyle adjustments. The emotional and mental weight of dealing with a long-term medical illness, along with the possibility of experiencing bleeding difficulties, emphasizes the need to take into account the overall effect of anticoagulation on patients' health and happiness [21].

Future Directions in Anticoagulation Research

Research is underway to create new anticoagulant drugs, which are changing the field of anticoagulation in MPE. The primary objective of these agents is to improve effectiveness, minimize the likelihood of bleeding, and streamline the medication administration process. Factor Xa inhibitors, such as anticoagulants, are being studied for their possible use in managing MPE [21]. The research focuses on optimizing anticoagulant methods to provide therapeutic benefits while reducing problems. Customizing anticoagulant treatments according to specific patient attributes, such as age, kidney function, and concurrent illnesses, can improve the accuracy and safety of long-term anticoagulation therapy. Individualized treatment strategies may become prevalent in the future of anticoagulation in MPE. These algorithms would consider individual patient characteristics, the likelihood of experiencing another blood clot, the propensity to bleed, and overall well-being in the long run. These customized methods strive to achieve a careful equilibrium between the effectiveness of therapy and the safety of the patient [21].

Comparative analysis of thrombolysis and anticoagulation

The treatment of MPE requires careful assessment of therapeutic options, with thrombolysis and anticoagulation emerging as crucial therapies. This section performs a thorough and detailed comparison of thrombolysis versus anticoagulation, using information from clinical trials and studies. In addition, we explore the essential element of patient stratification to identify the appropriate candidates for each therapy method. The evaluation of hemodynamic stability is a crucial aspect that affects the decisions made in the complex field of managing MPE. Table 2 provides a detailed comparison of key criteria between thrombolysis and anticoagulation, aiding decision-making considerations.

**Table 2:** Comparison of thrombolysis and anticoagulation strategies

Criteria	Thrombolysis	Anticoagulation
Hemodynamic stability	Requires careful assessment of stability	Typically, suitable for stable patients [13]
Bleeding risks	Increased risk, especially intracranial bleeding	Lower bleeding risk compared to thrombolysis [13]
Efficacy in thrombus resolution	Rapid thrombus dissolution	Prevents further thrombus propagation [16]
Long-term management	May require extended anticoagulation	Continuation of anticoagulation for prevention [20]

Clinical Trials and Studies

The PEITHO experiment is a groundbreaking study that has dramatically impacted the discussion on thrombolysis in PE. This study conducted a randomized controlled trial to assess the effectiveness and safety of tenecteplase in combination with heparin compared to a placebo in normotensive individuals with high-risk PE. The study showed that tenecteplase reduced the combined occurrence of death from any cause and hemodynamic decompensation. However, this benefit came with the drawback of an elevated risk of significant bleeding [21]. The guidelines established by the American College of Chest Physicians (ACCP) offer a thorough structure for the use of anticoagulation in treating VTE, including PE. For stable patients, it is recommended to use anticoagulation as the primary treatment approach, either with UFH or LMWH, followed by oral anticoagulants [22]. The guidelines emphasize the significance of personalized treatment choices, taking into account patient attributes and risk factors. Multiple randomized controlled trials (RCTs) and meta-analyses have enhanced our comprehension of the consequences of anticoagulation in MPE. The efficacy and safety of DOACs have been investigated in studies such as the EINSTEIN-PE study, the AMPLIFY trial, and the Hokusai-VTE trial compared to standard anticoagulation. These trials have yielded valuable information regarding the comparable or even better effectiveness of DOACs in specific groups of patients [23]. Ongoing research explores hybrid methods integrating thrombolysis with anticoagulation or employing catheter-directed therapies in particular instances. The SEATTLE II trial investigated the application of ultrasound-assisted catheter-directed thrombolysis in patients diagnosed with intermediate-risk PE. These novel approaches seek to maximize the effectiveness of therapy while limiting the potential dangers associated with each specific intervention.

Patient Stratification: Identifying the Right Candidates

Efficient categorization of patients is crucial in deciding the treatment strategy for MPE. Risk stratification methods, such as the PESI and the ESC risk assessment model, help classify patients into low, middle, and high-risk categories. These tools consider clinical characteristics such as age, comorbidities, and vital signs. They allow doctors to customize interventions based on the individual risk profile of each patient. Thrombolysis is especially relevant in high-risk situations of MPE, where patients show symptoms of hemodynamic compromise or are in immediate danger of cardiovascular collapse [23]. The PEITHO trial showed that tenecteplase can reduce the incidence of hemodynamic decompensation in normotensive patients with high-risk characteristics. It is essential to identify these individuals who are at high risk to start thrombolytic therapy promptly and effectively. When patients with intermediate-risk MPE show symptoms of right ventricular dysfunction but are not hemodynamically unstable, the choice between thrombolysis and anticoagulation is more complex [23]. Various patient-specific characteristics, such as bleeding risks, comorbidities, and preferences, determine the most suitable therapy approach. Recent studies have examined the practicality of risk-adapted approaches, which involve customizing interventions according to personalized risk evaluations. In cases of MPE with low risk, where patients have stable blood flow and do not have significant issues with the right ventricle, the conventional treatment approach is anticoagulation [24]. The ACCP guidelines endorse the utilization of anticoagulants in these situations, highlighting the safety and effectiveness of this approach. In this group, the attention is directed at avoiding the further growth of blood clots and reducing the chances of their recurrence.

Assessing Hemodynamic Stability

Evaluating the integrity of blood circulation is crucial in determining the appropriate treatment approach for MPE. Heart rate, blood pressure, and respiration rate are necessary clinical measures that provide valuable information about the patient's cardiovascular condition. Biomarkers such as troponins and brain natriuretic peptide (BNP) offer supplementary insights into the load on the right ventricle and damage to the myocardium. Incorporating these factors helps categorize the risk level and guides the decision-making process between using thrombolysis or anticoagulation. Advanced imaging techniques, specifically computed tomography pulmonary angiography (CTPA) and echocardiography, are crucial in evaluating hemodynamic stability [24]. CTPA offers comprehensive data regarding the size and position of pulmonary emboli, assisting in assessing risk levels. Echocardiography, with a specific focus on evaluating the function of the right ventricle, provides essential information for the comprehensive assessment of the circulatory system. Integrating imaging findings with clinical factors enhances the comprehension of the patient's cardiovascular condition. The state of hemodynamic stability is not fixed, and it is essential to employ dynamic risk assessment to adjust therapeutic measures as needed. The continuous monitoring of clinical indicators, biomarkers, and imaging data enables the detection of developing clinical situations [24]. A comprehensive and flexible strategy guarantees that treatment actions align with the patient's evolving hemodynamic profile, maximizing results and reducing avoidable hazards. Evaluating hemodynamic stability necessitates collaborating among specialists, including cardiologists, pulmonologists, intensivists, and other pertinent experts. This collaborative method guarantees a comprehensive assessment of the patient's condition, integrating many viewpoints and specialized knowledge. The consensus-driven decision-making process improves the accuracy of therapeutic interventions by aligning with each patient's specific requirements.

Future Directions in Comparative Analysis

Thrombolysis techniques are expected to develop in future research, emphasizing improving fibrinolytic drugs, minimizing bleeding risks, and investigating new administration regimens. Thrombolysis refinement aims to improve the treatment's effectiveness while reducing the likelihood of problems, resulting in a more advantageous balance between risks and benefits. Progress in anticoagulation research may entail customizing approaches according to individual patient characteristics, pharmacogenomics, and the application of innovative anticoagulant substances [25]. The current investigation of factor Xa inhibitors and other advanced anticoagulants seeks to offer choices that possess enhanced safety profiles and therapeutic accuracy. In the future, the field of MPE management may see the emergence of tailored treatment algorithms that effectively combine risk categorization, patient characteristics, and developing hemodynamic assessments. These algorithms would assist doctors in navigating the intricate decision-making process, enhancing outcomes by utilizing personalized therapeutic techniques [25].

Trade-offs and complications

The complex nature of managing MPE is characterized by the selection of treatment options and the inherent trade-offs and consequences associated with these therapies. This section explores the complex factors related to the risks of bleeding associated with thrombolysis, the enduring effects of anticoagulation, and the subtle influence on patients' quality of life. Clinicians must thoroughly comprehend these trade-offs to effectively navigate the intricate decision-making process and maximize results for persons dealing with the significant consequences of MPE.

Bleeding Risks with Thrombolysis

Thrombolysis, being a powerful fibrinolytic technique, carries the inherent risk of bleeding consequences. Agents such as alteplase or tenecteplase can trigger systemic fibrinolysis, which goes beyond the pulmonary vasculature and may cause bleeding in different body areas. Intracranial bleeding is a severe worry, as it has the potential to have life-threatening consequences. The careful selection of patients and continuous monitoring are necessary to maintain a delicate balance between achieving thrombus clearance and minimizing bleeding risks [25]. Determining which patients are more susceptible to bleeding is crucial in reducing problems related to thrombolysis. Factors such as age, comorbidities, previous bleeding history, and simultaneous use of anticoagulants or antiplatelet medications have an impact on the risk of bleeding. By incorporating risk stratification measures, like the HAS-BLED (Hypertension, Abnormal Renal/Liver Function, Stroke, Bleeding History or Predisposition, Labile INR, Elderly, Drugs/Alcohol Concomitantly) score, into the decision-making process, it becomes easier to evaluate the total risk profile and customize treatment options to reduce the chances of bleeding [26]. Cerebral hemorrhage is a severe and potentially deadly consequence of thrombolysis. The heightened risk is attributed to the permeability of the blood-brain barrier and the susceptibility of cerebral arteries. Choosing suitable patients becomes even more critical in reducing this risk by carefully considering contraindications such as recent surgery inside the skull, ongoing bleeding, or existing brain blood vessel abnormalities [26]. To minimize the dangers of bleeding that are connected with thrombolysis, many additional treatments might be utilized. The purpose of administering antifibrinolytic drugs, such as tranexamic acid, is to weaken the process of systemic fibrinolysis and decrease the likelihood of bleeding. Furthermore, it is crucial to monitor vital signs closely, do sequential imaging, and regularly evaluate coagulation markers to identify bleeding indications and take immediate action rapidly.

Long-Term Consequences of Anticoagulation

The enduring ramifications of anticoagulation extend beyond the immediate stage of managing MPE, as long-term treatment brings forth its own distinct set of factors to be considered. Extended administration of anticoagulant medication, which is sometimes required to prevent the recurrence of blood clots, increases the likelihood of experiencing bleeding issues. Possible outcomes of this condition include bleeding in the gastrointestinal tract, urinary system, and inside the skull. To manage this, it is essential to find the right balance between using anticoagulant therapy and reducing the risk of bleeding [26]. The correlation between extended use of anticoagulant medication, specifically vitamin K antagonists such as warfarin, and negative impacts on bone health has attracted significant attention. Anticoagulants disrupt the process of vitamin K metabolism, which can reduce bone mineral density and an elevated susceptibility to osteoporosis. The consequences for the risk of fractures and the overall health of the musculoskeletal system should be taken into account, particularly in populations with a pre-existing vulnerability to bone-related disorders. Some anticoagulants, including DOACs, can affect kidney function. Individuals with kidney problems may face difficulties in maintaining the appropriate levels of anticoagulation treatment. This may necessitate modifications in dosage or the use of different anticoagulant methods [27]. The effect on kidney function highlights the importance of frequent monitoring and a customized strategy for administering anticoagulant medication in persons with impaired renal function. The enduring ramifications of anticoagulation extend beyond physiological measures, influencing the overall quality of life for persons with MPE. The onus of long-term treatment, which entails frequent surveillance, dietary limitations (in the case of warfarin), and possible adverse reactions, can affect patients' day-to-day activities [28]. Ensuring a balance between the essential requirement for anticoagulation and its potential effects on quality of life is crucial in collaborative decision-making between healthcare professionals and patients.

Impact on Patient Quality of Life

In addition to the physiological factors, it is crucial to acknowledge the psychosocial consequences of thrombolysis and anticoagulation. The severity of an MPE diagnosis, combined with the difficulties related to rigorous therapy, can lead to increased anxiety, depression, and overall psychological distress in affected persons. Healthcare providers should embrace a comprehensive approach that considers patients' overall mental and social well-being at every stage of their treatment. Certain anticoagulants, namely those that impact the vitamin K pathway, have been linked to potential cognitive consequences [28]. Warfarin has been examined closely for its potential influence on cognitive function. Although continuous research is being conducted in this field, it is essential to address the concerns regarding cognitive decline and the broader effects on neurocognitive health, particularly in populations that are susceptible to cognitive impairment. Patient compliance with long-term anticoagulant treatment is a notable difficulty. The complexities of modifying doses, adhering to dietary restrictions, and the necessity of consistent monitoring contribute to the intricacy of adherence. Adherence may be further complicated by factors such as forgetfulness, the burden of taking multiple pills, and anxiety about the hazards of bleeding. Facilitating adherence to anticoagulant regimes requires including patient education, individualized assistance, and integrating patient preferences into therapeutic decisions [28]. Open communication between physicians and patients is crucial for shared decision-making in managing MPE, as it helps navigate the complexities and trade-offs involved. It is essential to consider patient preferences, values, and goals while customizing therapy strategies. Collaborative decision-making enables patients to engage in their healthcare actively, providing autonomy and encouraging treatment adherence.

Future Directions in Addressing Trade-Offs

Emerging developments in bleeding risk prediction models are imminent to enhance risk evaluations and inform treatment choices. Integrating genetic factors, biomarkers, and advanced imaging techniques into predictive models can improve bleeding risk evaluation accuracy. The advancement of tailored methods for predicting the risk of bleeding shows potential in optimizing the trade-off between the effectiveness of treatment and safety [29]. In the future, researchers may investigate tailored anticoagulation techniques that seek to reduce the chances of bleeding while maintaining the effectiveness of the treatment. Customizing anticoagulation treatments according to unique patient attributes, such as genetic predispositions and coexisting medical conditions, can enhance long-term anticoagulation by balancing risks and benefits. To acknowledge the psychological and social consequences of MPE and its treatment, future efforts could concentrate on incorporating psychosocial support initiatives into regular healthcare practices. Including mental health experts, patient support groups, and educational materials can enhance comprehensive patient care by addressing physical and psychological well-being [29].

Decision-making factors in MPE management

MPE is a challenging medical situation that requires careful decision-making to get the best possible outcomes for the patient. This section explores the several complex aspects that affect decision-making in the management of MPE. These factors include considering patient-specific considerations, following guidelines and recommendations, and recognizing the critical role of collaborative decision-making in clinical practice. Table 3 highlights the characteristics of commonly used risk stratification tools in MPE management.

**Table 3:** Risk stratification tools PESI: Pulmonary Embolism Severity Index; ESC: European Society of Cardiology

Tool	Parameters Assessed	Risk Categories
PESI	Age, comorbidities, vital signs	Low, intermediate, high-risk groups [22]
ESC risk assessment model	Biomarkers, vital signs, imaging findings	Low, intermediate, high-risk categories [25]

Incorporating Patient-Specific Factors

Individual patient characteristics are crucial in customizing treatment approaches for MPE. The risk-benefit profile of therapies is strongly influenced by age and comorbidities, such as cardiovascular disease, renal impairment, or bleeding tendencies. Geriatric patients present distinct obstacles, necessitating a meticulous equilibrium between therapy effectiveness and the reduction of potential consequences. Risk stratification techniques, such as the PESI and the ESC risk assessment model, provide a methodical way to classify patients according to their risk profiles. By integrating these tools into decision-making, doctors can effectively identify high-risk individuals who might benefit from more intensive therapies such as thrombolysis. At the same time, they can also consider the specific needs of patients at low and intermediate risk [29]. Evaluating the stability of the circulatory system is a fundamental aspect of making decisions on managing malignant pleural effusion. Patients who have stable blood circulation may be suitable for regular anticoagulation treatment. However, patients who show signs of compromised blood circulation or cardiovascular failure require more intensive measures such as thrombolysis or surgical embolectomy [30]. Continual monitoring and dynamic risk assessments guide the ongoing modification of treatment strategies according to the patient's changing clinical condition. The presence of contraindications, such as recent surgeries, active bleeding, or pre-existing conditions, affects the choice of therapeutic approaches. It is crucial to consider the contraindications and preferences of each patient while making decisions. Active involvement of patients in shared decision-making guarantees that their values, concerns, and treatment objectives are thoroughly considered during the decision-making process [30]. Pharmacogenomics advancements provide an understanding of the differences in drug response among individuals. Genetic characteristics that affect medication metabolism and how individuals respond to anticoagulants can help inform individualized therapy approaches. Customizing therapies according to an individual's genetic profile has the potential to enhance therapeutic effectiveness while reducing adverse side effects.

Guidelines and Recommendations

The guidelines established by the ACCP play a fundamental role in providing accurate information for making evidence-based decisions regarding the therapy of malignant pleural effusion. These guidelines offer extensive suggestions regarding anticoagulation methods, thrombolysis, and the significance of surgical embolectomy. Following these standards guarantees a consistent and fact-based approach, especially when there are no reasons to avoid them or specific factors to consider. The guidelines from the ESC supplement the ACCP recommendations, offering a European viewpoint on the management of MPE [31]. These guidelines take into account the differences in healthcare practices between regions and provide a further understanding of risk stratification, diagnostic algorithms, and treatment strategies. Clinicians must adhere to both sets of rules to ensure a comprehensive and well-informed approach to decision-making on a global scale. The process of making decisions in MPE management is constantly changing as standards adapt to include new and emerging evidence. These guidelines are always influenced by ongoing research and the results of clinical trials. Healthcare professionals must remain updated to ensure that their decision-making aligns with the most recent study, promoting a culture of ongoing learning and adjustment to the changing landscape of managing MPE [31].

Shared Decision-Making in Clinical Practice

Shared decision-making is a cooperative, patient-focused method that involves transparent communication between medical professionals and patients. It recognizes individual patients' distinct viewpoints, principles, and choices, actively engaging them in making decisions. Shared decision-making in MPE care improves patient autonomy, satisfaction, and treatment adherence, especially when therapeutic choices have essential consequences [31]. Integrating patient viewpoints into decision-making starts with providing thorough patient education. It is necessary to offer precise and understandable information on the characteristics of MPE, the therapeutic choices that are accessible, the related hazards, and the prospective results. By involving patients in decision-making, informed consent enables them to actively engage in choices that align with their objectives and principles [32]. Efficient communication between clinicians and patients is crucial for collaborative decision-making. Medical professionals must effectively communicate intricate medical information that is easily understandable and accessible to patients. Promoting an atmosphere that motivates patients to voice their concerns, inquire about matters, and actively participate in conversations cultivates a cooperative process of decision-making [32]. Decision aids, such as educational booklets, online resources, or interactive technologies, can promote shared decision-making by offering patients supplementary resources to augment their comprehension. These aids can enhance clinician-patient conversations and enable individuals to make well-informed decisions that align with their beliefs and preferences.

Future directions and research needs

The future of MPE management shows potential with continuous advancements in therapeutic strategies. Current research is investigating new substances that can dissolve blood clots (fibrinolytic drugs), tactics that specifically target the prevention of blood clot formation (anticoagulation strategies), and interventions that combine clot-dissolving substances with catheter-directed procedures. These developments strive to maximize the effectiveness of treatments while limiting potential hazards, offering doctors a wide range of tools to customize interventions based on unique patient requirements. Multiple ongoing clinical trials are now addressing essential research gaps and improving decision-making in the management of MPE. Trials like PEITHO-2 examine the function of thrombolysis in pulmonary embolism with intermediate risk, while other trials study the effectiveness and safety of new anticoagulant medications [33]. It is crucial to monitor the results of these trials to integrate the most recent evidence into therapeutic practice. Although there have been breakthroughs, there are still unresolved issues in the administration of MPE.

Further investigation is needed to anticipate the likelihood of bleeding accurately, customize therapies based on genetic factors, and address the psychological and social consequences of MPE. Future research should prioritize fixing these limitations to improve the comprehensiveness and precision of decision-making [34]. The incorporation of artificial intelligence (AI) into decision-making processes has the potential to enhance risk assessments and customize solutions. AI algorithms can evaluate large volumes of data to predict how particular patients will respond to treatment. These algorithms may also help optimize assessments of the risks and benefits associated with different treatment options and guide tailored treatment plans. Investigating the potential uses of AI in multiple project environment management is a promising area for future research [35].

## Conclusions

To summarize, the investigation into the management of MPE has revealed significant findings that have important implications for clinical practice. The key findings emphasize the significance of adopting a sophisticated approach that considers individual patient variables, adheres to changing rules, and embraces collaborative decision-making. Optimizing results relies on achieving a delicate equilibrium between thrombolysis and anticoagulation, which is informed by risk stratification and a comprehensive understanding of bleeding risks. A broad perspective is required when making decisions due to the psychological and social effects of MPE, the enduring impacts of anticoagulation, and the dangers of bleeding associated with thrombolysis. These findings directly affect clinical practice, emphasizing the need for physicians to incorporate a patient-centered approach, stay updated on new recommendations, and encourage open communication with patients. The future of MPE management hinges on continuous advancements, encompassing evolving therapeutic methodologies, ongoing clinical studies, and the incorporation of artificial intelligence. These developments have the potential to bridge existing gaps and enhance the process of decision-making. Future research should prioritize doing comprehensive studies on bleeding risk prediction models, implementing focused anticoagulation methods, and developing therapies that address the psychosocial elements of MPE. Continually examining new evidence and incorporating innovative technologies will improve the accuracy and thoroughness of decision-making in this ever-changing field.
